# Synthesis of cyclic carbonates from epoxides and carbon dioxide catalyzed by talc and other phyllosilicates

**DOI:** 10.1186/s13065-020-00713-2

**Published:** 2020-10-20

**Authors:** Fiona Nakibuule, Steven Allan Nyanzi, Igor Oshchapovsky, Ola F. Wendt, Emmanuel Tebandeke

**Affiliations:** 1 Department of Chemistry, College of Natural Sciences, Makerere University, P. O. Box 7062, Kampala, Uganda; 2 Centre for Analysis and Synthesis, Department of Chemistry, Lund University, P.O. Box 124, 221 00 Lund, Sweden; 3 Department of Inorganic Chemistry, Ivan Franko National University of Lviv, Kyryla i Mefodiya Str. 6, Lviv, 79005 Ukraine

**Keywords:** Carbon dioxide, Cycloaddition, Cyclic carbonates, Epoxides, Talc, Phyllosilicates

## Abstract

Naturally occurring phyllosilicate minerals such as talc and vermiculite in conjunction with n-tetra butyl ammonium bromide (TBAB) co-catalyst were found to be efficient in the coupling of CO_2_ with epoxides to form cyclic carbonates. The reaction was carried out in a pressurized autoclave reactor at moderate pressures of 10–35 bars and temperatures of 100–150 °C. The optimized catalyst system exhibited > 90% conversion of the epoxides and > 90% selectivity for the desired cyclic carbonates, in the presence or absence of a solvent. The selectivity of the catalytic system could be improved with heat pre-treatment of the phyllosilicates albeit this resulted in slightly lower epoxide conversion. The results obtained using the heat treated phyllosilicates strongly support the hydrogen bond assisted mechanism for the cycloaddition of epoxides and CO_2_. The cycloaddition reaction could also be carried out in the absence of TBAB, although lower cyclic carbonate yields were observed. The phyllosilicate part of the catalyst system is heterogeneous, easy to separate after completion of reactions and reusable a number of runs without loss of activity.

## Introduction

**Scheme 1:**
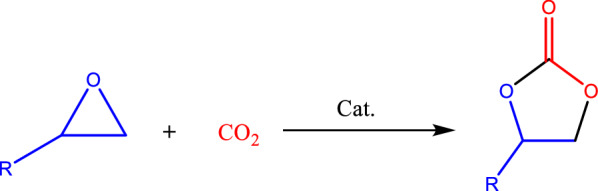
Cyclic carbonate synthesis

Modern societies today are highly dependent on carbonaceous fossil fuels as the primary source of energy [1–3]. Complete combustion of these fuels produces carbon dioxide (CO_2_), a gas whose concentration in the atmosphere has increased significantly: and is believed to contribute significantly to global warming [4, 5]. There are increasing concerns for global warming and heightened interest worldwide to reduce CO_2_ atmospheric emissions. One of the solutions being considered to this problem is converting CO_2_ into chemical products for which there is significant commercial demand [6–8]. Although the amount of CO_2_ that can be consumed through chemical production is small compared to the amount generated by fossil fuel combustion, its conversion nevertheless would be favorable from the green chemistry perspective. Moreover, if done efficiently, using CO_2_ as a chemical feedstock, would constitute a net positive contribution towards sustainability [9–11]. Unfortunately, due to the thermodynamic stability of CO_2_ its activation and insertion into organic molecules still remains a challenge. A promising process in CO_2_ chemical fixation, is its insertion into epoxides to produce cyclic carbonates (Scheme 1).

The CO_2_ and epoxide cycloaddition reaction, proceeds with 100% atom economy, and thus constitutes one of the most efficient examples of artificial CO_2_ fixation. Moreover, this approach of cyclic carbonate production is environmentally benign compared to the traditional syntheses, which involve use of the highly toxic and corrosive phosgene [12]. Cyclic carbonates have found extensive use as excellent aprotic polar solvents, electrolytes in secondary batteries, monomers in the production of polycarbonates, chemical ingredients in preparation of medicines or agricultural chemicals, and other applications [13–15].

Several industrial processes for cyclic carbonate production from CO_2_ employing homogeneous catalysts have been reported [15–17]. Although a number of these catalytic systems exhibit high efficiency, the catalysts are undesirably dissolved in the phase containing the cyclic carbonates, creating separation and purification challenges at the end of the process. In contrast, inorganic heterogeneous catalysts tested in the cycloaddition process offer some advantages such as ease of post reaction separation, recyclability and high thermal stability. Indeed, various inorganic heterogeneous catalysts, such as Mg–Al mixed oxides [18], basic metal oxides [19–21], iron-based composite [22], smectites [23, 24], and zeolites [25, 26], have been reported for this process. Unfortunately, several of these solid catalysts have insufficient activity and most of them require additives to ensure reasonable conversion and selectivity. Thus, research in the development of low cost, thermally stable and efficient heterogeneous catalysts for coupling of CO_2_ and epoxides, is still gaining considerable interest. Taking into account that some silicate-based catalysts like zeolites [25, 26] and smectites [23, 24] have demonstrated promising catalytic activity in the CO_2_ and epoxide cycloaddition reaction under mild conditions, further testing and exploration of this group of minerals is highly desirable. These materials have advantages including: natural occurrence, low cost, their synthetic analogues are easy to prepare; and the catalyzed reaction proceeds in heterogeneous phase, hence offering easy catalyst separation and recyclability [25, 26].

The smectites belong to the phyllosilicate group of silicates, which includes minerals such as talc, vermiculite, biotite, phlogopite, among others [27]. Most of the minerals in this group are layered and are comprised of octahedral and tetrahedral sheets. The octahedral sheets contain divalent or trivalent cations such as Mg^2+^ and Al^3+^ surrounded by six oxygen atoms, whereas the tetrahedral sheets contain Si^4+^ cations surrounded by four oxygen atoms. Generally, most of the phyllosilicates are OH bearing in their structures and contain varying amounts of other metal cations. The presence of numerous hydroxyl groups and active metal cations such as Mg^2+^, Al^3+^, Fe^2+^ in the structures of the phyllosilicates strongly suggests that they have catalytic potential in the cycloaddition reaction; particularly considering the hydrogen bond assisted mechanism of CO_2_ and epoxide coupling [22]. A number of studies have indicated that hydrogen bonding between the catalyst and substrate plays a key role in enhancing reactions [28]. Indeed, catalytic materials rich in hydroxyl groups have proved to be efficient hydrogen bonding catalysts [29–31]. Qu and co-workers [22], reported that hydrogen bonding at the solid/liquid interface can activate epoxides and stabilize ring opening intermediates. It has also been reported that through hydrogen bonding, small amounts of water can assist a Lewis base catalyzed reaction between an epoxide and CO_2_ [32]. Further on, studies have shown that the weakly acidic surface silanol groups can activate propylene oxide, which subsequently reacts with a nucleophile to open the epoxide ring [33]. A combination of the previous observations motivated this investigation into the performance of phyllosilicate minerals as catalysts for cyclic carbonate synthesis.

Herein we report an efficient catalytic system for the phosgene-free conversion of CO_2_ into cyclic carbonates catalyzed by talc, vermiculite and other phyllosilicates, in conjunction with tetra butyl ammonium bromide (TBAB). The CO_2_ and epoxide coupling process was carried out without addition of TBAB co-catalyst in some experiments, albeit with lower yields of cyclic carbonates. The phyllosilicates contain several hydroxyl groups to facilitate the coupling of CO_2_ and epoxides, and the results of this study, strongly support the hydrogen bond assisted mechanism of the cycloaddition process. The phyllosilicates used in this study are naturally occurring, and some of them were sampled from deposits in Uganda. The low cost and natural abundancy of silicate and alumina minerals, coupled with their unique properties make them valuable in several catalytic applications [34].

## Results and discussion

### Elemental composition of talc and vermiculite

The elemental composition data for talc used in this study is presented in Table 1. The results show presence of significant amounts of Si, Mg, Al and Fe, whereas other elements such as Ca, K, Na, Mn and Cr were detected in low amounts. These compositions are consistent with the reported structure of talc [27]. Talc is mainly comprised of Mg in the octahedral sheet and Si in the tetrahedral sheets; the presence of other elements such as Al and Fe suggests tetrahedral or octahedral substitutions, for example Fe normally substitutes for Mg in the octahedral layer [35].

**Table 1:** Elemental composition of talc and vermiculite

Entry	Element expressed as oxide	Talc composition [wt.%]	Vermiculite composition [wt.%]
1	SiO_2_	54.28	42.98
2	Al_2_O_3_	2.48	14.24
3	Fe_2_O_3_	6.07	7.13
4	MgO	29.35	21.35
5	CaO	1.22	2.06
6	MnO	0.26	0.18
7	K_2_O	0.12	0.92
8	Na_2_O	0.25	0.48
9	Cr_2_O_3_	0.08	0.04
Sum*		94.1	89.4

*Sum excluding volatiles such as water.

The observed chemical composition of talc is comparable to that reported for naturally occurring talc deposits from Lamal Pougue, Cameroon [36]. However, it should be noted that every talc deposit is unique with regard to chemistry and morphology due to variations in geological formation [27]. The elemental composition data for the vermiculite sample used in the study presented in Table 1, show significant amounts of Si, Mg, Al, Fe and Ca; other elements such as K, Na, Mn and Cr were also detected in low amounts. These observations are consistent with the structure of vermiculite [27], and is comparable with the reported chemical composition of naturally occurring deposits [37, 38].

### Infrared spectroscopy of the talc catalyst

The IR spectrum of the talc material is shown in Additional file 1: Figure S1. The spectrum shows peaks at 451 and 469 cm^−1^ which are attributed to Mg–O–Si and Si–O–Si bending vibrations, respectively. The sharp absorption at 669 cm^–1^ is attributed to the bending vibration of Mg-OH in the talc structure. The absorption band at 1014 cm^−1^ is due to stretching vibration of Si–O of the [SiO_4_] tetrahedra [39, 40]. However, the observed intensities of some bands were relatively weak compared to those reported in previous studies [35, 41, 42]. This could be attributed to structural degradation of the octahedral and tetrahedral sheets under mechanical stress of grinding, and possibly lower content of OH groups in the pristine mineral. Some bonds in the talc structure such as Mg–O and Mg–OH may break during grinding, resulting in disruption of linkages between octahedral and tetrahedral sheets [41].

### BET studies of the talc catalyst

The BET specific surface area (S_BET_) for the talc catalyst was 2.78 m^2^/g, with a pore size of 296.7 Å and a mesoporous pore volume of 0.02 cm^3^/g. The microporous pore volume was negligible (≤ 0.0003 cm^3^/g). The N_2_ adsorption–desorption isotherm of the talc catalyst as shown in Additional file 1: Figure S2 is characterized by a Type IV isotherm with a narrow hysteresis that spreads across part of the plateau region of the adsorption curve, characteristic of materials with slit-shaped pores [43]. The isotherm obtained for talc in this study, is similar to those reported for talc and other mesoporous materials by Sprynskyy and co-workers [44].

### Powder X-ray diffraction of talc

**Fig. 1:**
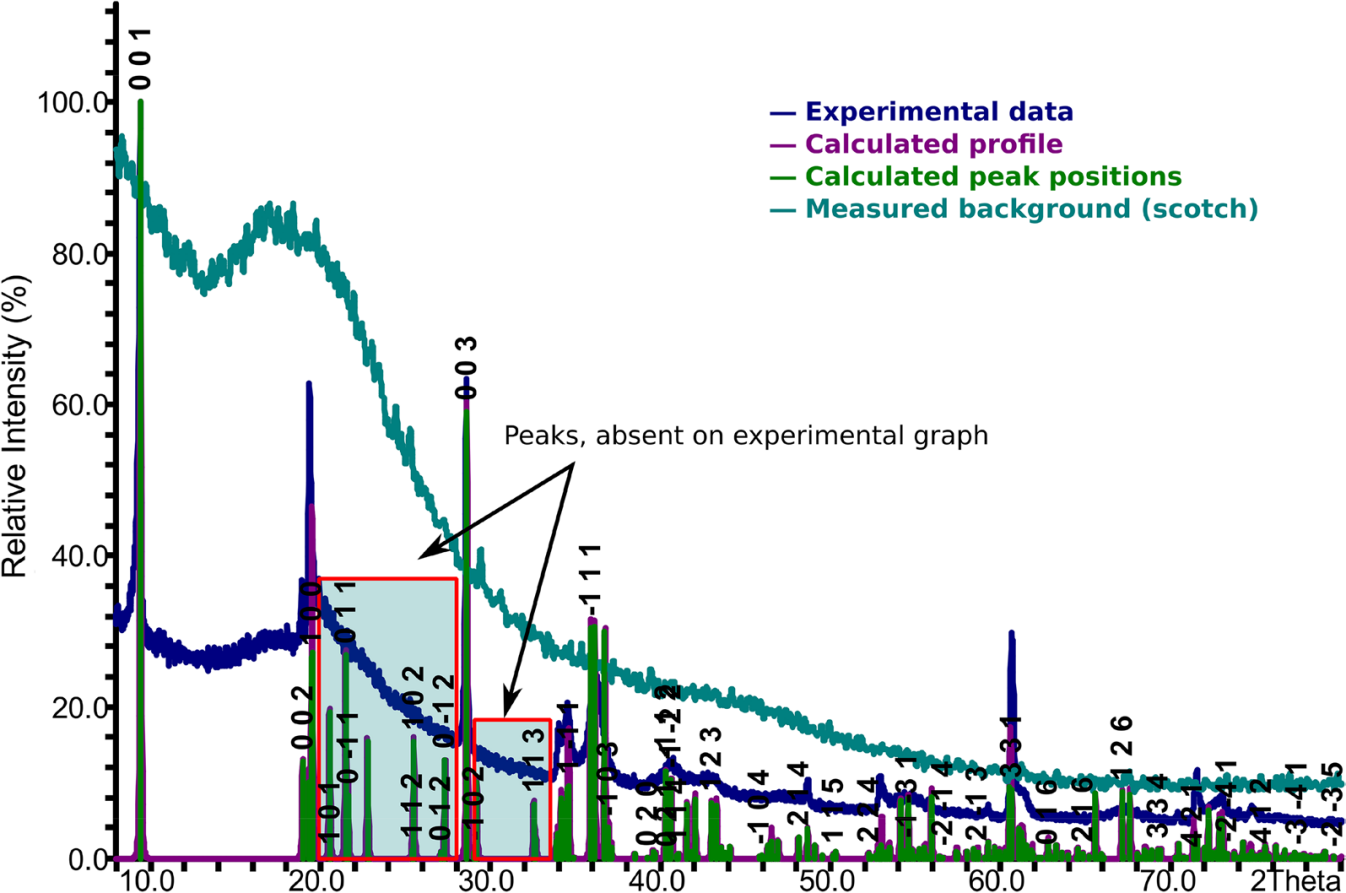
The experimental powder XRD pattern for talc and the calculated pattern from the completely ordered structure model [45]

The powder X-ray diffraction pattern of the talc catalyst is shown in Fig. 1. The relative intensity and peak positions of the XRD pattern are in agreement with the typical structure of talc. The XRD data show the 2θ and crystal plane (hkl) values of three important peaks of talc including 2θ_001_ = 9.40°, 2θ_002_ = 19.90°, and 2θ_003_ = 28.90°. The XRD peaks are similar to the ones reported for the E-4 Emirdağ samples (actinolite 5% and talc 95%) by Ersoy and co-workers [42], but without the actinolite peak (2θ = 10.90°). A comparison of the experimental pattern, with the theoretically calculated one from the fully ordered model according to Perdikatsis and Burzlaff [45], also shows similarities in the structure albeit a few discrepancies, such as absence of some peaks between 20° and 35° due to structural disorder. The obtained data is also in agreement with the results reported by Kumar and co-workers [46] for milled talc samples. In general, positions of the strongest peaks coincide, but there are some absent peaks and shoulders near existing peaks. The shape of the peaks between 15° and 40° could be attributed to structure distortion usually caused by conditions of growth and/or metamorphization of the mineral. In addition, the distortion can be due to effects of grinding that could cause progressive structural disorder and subsequent amorphization of crystallites [41]. It is also common that during intensive grinding, the XRD basal 001 peak of talc decreases in intensity, whereas the 002 peak may disappear completely and an increase in back-ground in the 2θ = 15°–40° due to the formation of amorphous material is also expected [41]. Another important observation made is the similarity of the XRD pattern to the one obtained by Kogure and co-workers [47] in which the authors suggested that such a pattern is typical for silicates with extensive stacking disorder. Moreover, the peaks, absent on the experimental XRD pattern but existing on the theoretical pattern of the fully ordered model [45], further suggests stacking faults in the talc sample, as the same peaks are also absent in powder patterns of pristine talc samples in previous studies [41, 42, 47].

**Fig. 2:**
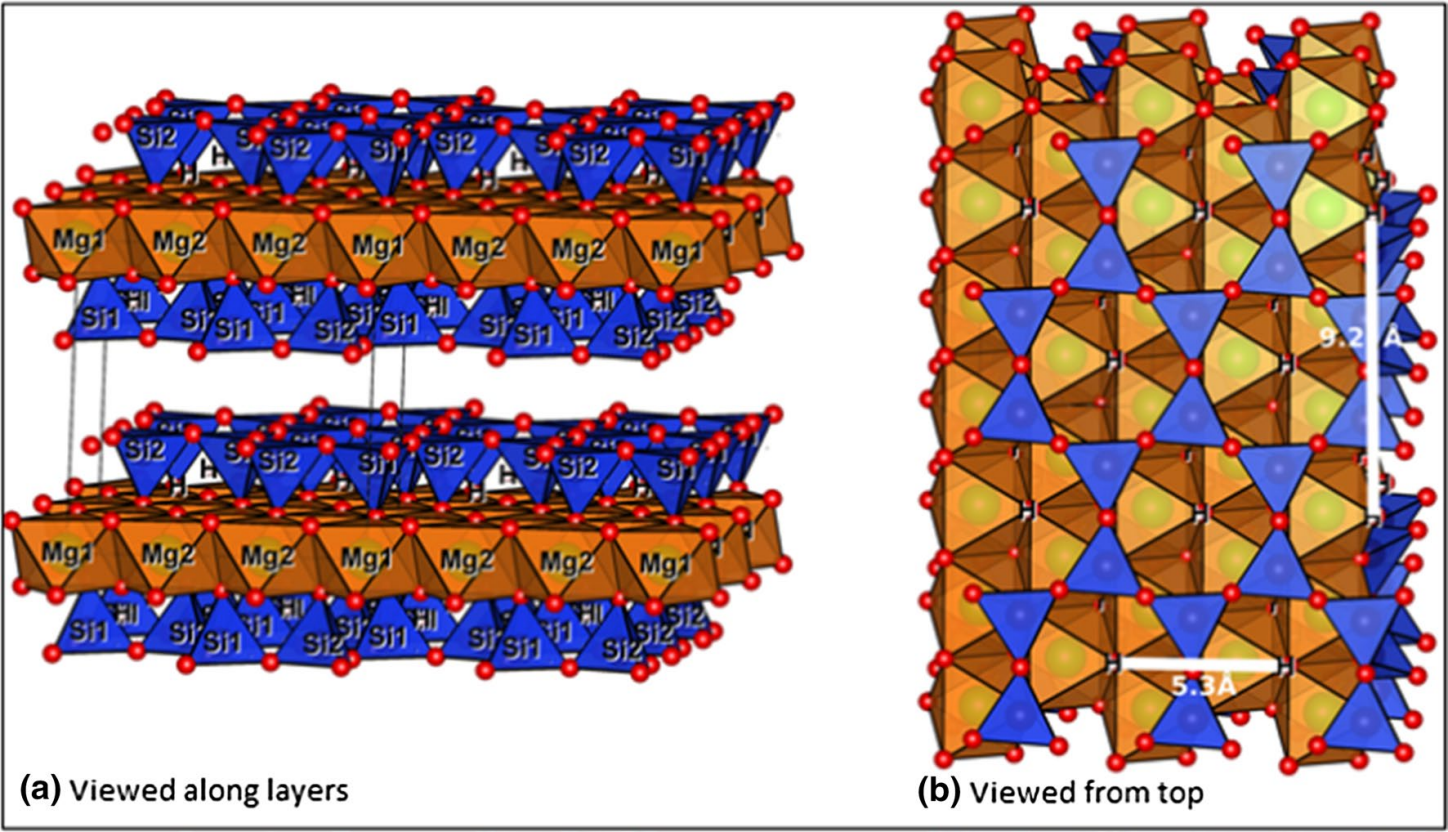
Simplified structure of talc showing the 2:1 layers generated using crystallographic data taken from Perdikatsis and Burzlaff [45]

The elemental composition, IR spectroscopy and powder X-ray diffraction results obtained for talc in this study, are all consistent with the reported structure [27]. The structure of talc Mg_3_(OH)_2_Si_4_O_10_ consists of alternating layers of vertex-sharing [SiO_4_] tetrahedra and corner-sharing [MgO_6_] octahedra. Two tetrahedral layers sandwich one octahedral layer through shared oxygen atoms, to form a 2:1 tri-layer, as shown in Fig. 2 which was generated using crystallographic data from Perdikatsis and Burzlaff [45]. These tri-layers are weakly connected to each other (Fig. 2a); which explains why inter- and intra-layer stacking faults and formation of different polytypes are possible. The [SiO_4_] tetrahedra in the undisturbed talc structure form hexagonal rings (Fig. 2b) around the hydroxyl groups that are attached to the [MgO_6_] octahedra as magnesol (–Mg–OH) groups. These sites are favourable for adsorption since a high coordination number of the adsorbed reactant is possible. The adsorbate can form up to 6 bonds to oxygen atoms: 6 bonds to silicon atoms and one hydrogen bond to the hydrogen from the hydroxyl group. The approximate radius of such adsorption site is 1.125 Å, taking into account the largest distance between oxygen atoms and their van der Waals radii. But the adsorbate does not need to go inside the hexagonal rings and can stay on top. There are two shortest characteristic distances between such an adsorption sites on the layer: 5.3 and 9.2 Å (Fig. 2b), but they do not match any characteristic distances in the epoxide and CO_2_. This implies that reactants will be adsorbed more likely only on the high-coordination site, but formation of bonds with other low-coordination sites is also possible.

In other words, the outer surface of layers could be considered as the most probable sites for catalysis. Other potential catalytic sites such as steps, defects, edges of layers and the region between two truncated hexagonal rings on the edge of crystal (Fig. 2a) could also be active due to the availability of several functional groups. Moreover, when the talc layers are disturbed during grinding, a diversity of functional groups are exposed. Since these were in the interior of the crystal, the valencies of these new functional groups are not completely saturated potentially causing them to be very reactive (vide infra) [48].

### Powder X-ray diffraction of vermiculite

**Fig. 3:**
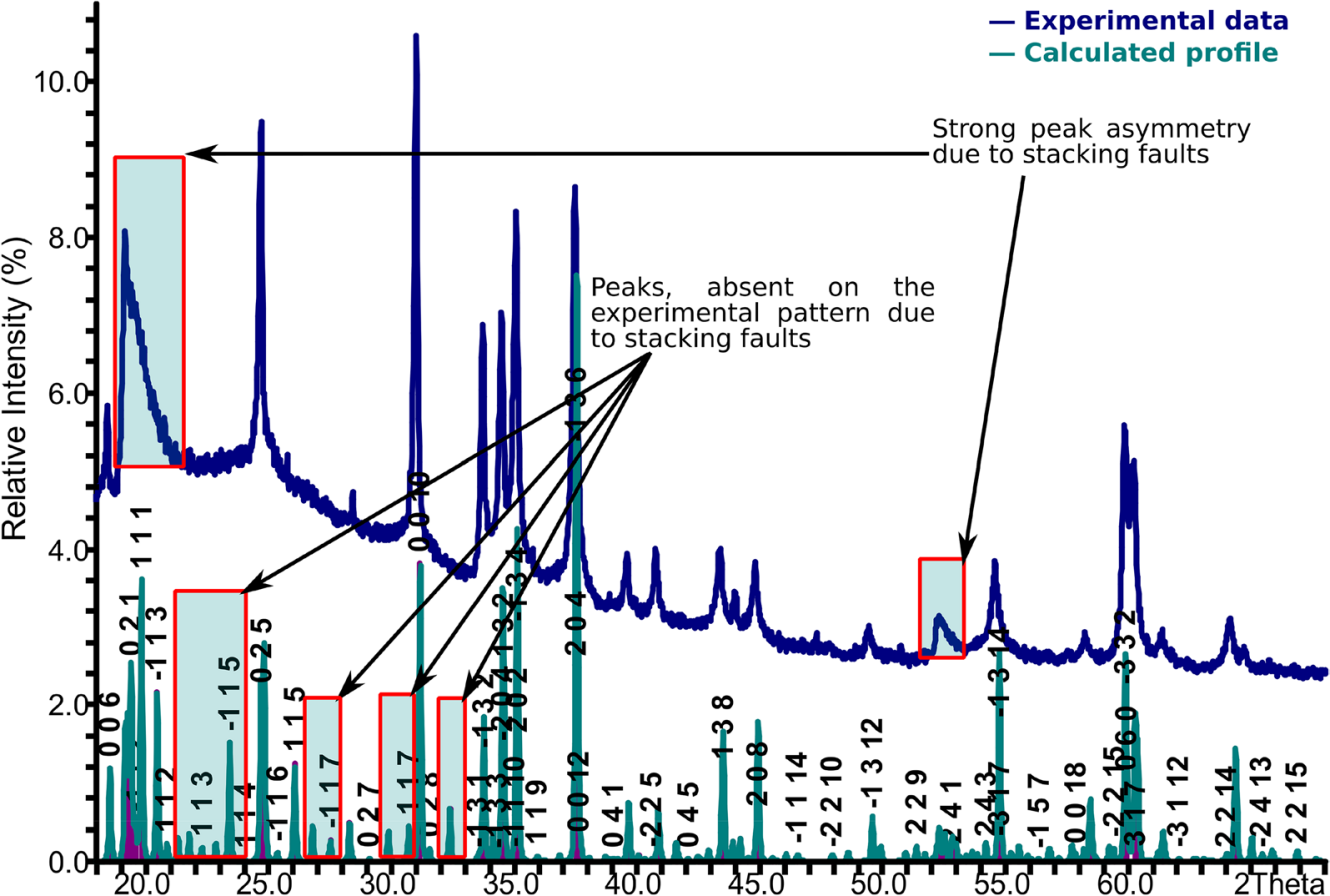
The experimental powder XRD pattern of vermiculite and the theoretical pattern calculated using data from Shirozu and Bailey [49]

The powder XRD pattern for the vermiculite sample is shown in Fig. 3; generally, the relative intensity and peak positions of the XRD pattern are in agreement with reported structures of vermiculite [37, 38, 49]. The pattern exhibited three small unindexed peaks at 28.4°, 43.96° and 64.7°, which possibly belong to traces of other minerals present in the sample. Among the conventional crystal structure models, which satisfactorily describe the observed experimental pattern, the model by Shirozu and Bailey [49] is the closest one. A comparison of the XRD pattern with those from previous studies show that the sample has a structure with stacking faults as observed for a vermiculite from Santa Olalla, Spain [37]. However, the absence of some reflections together with the strong asymmetry of the peak near 20° cannot exclude possible grinding effects which may cause progressive structural disorder.

**Fig. 4:**
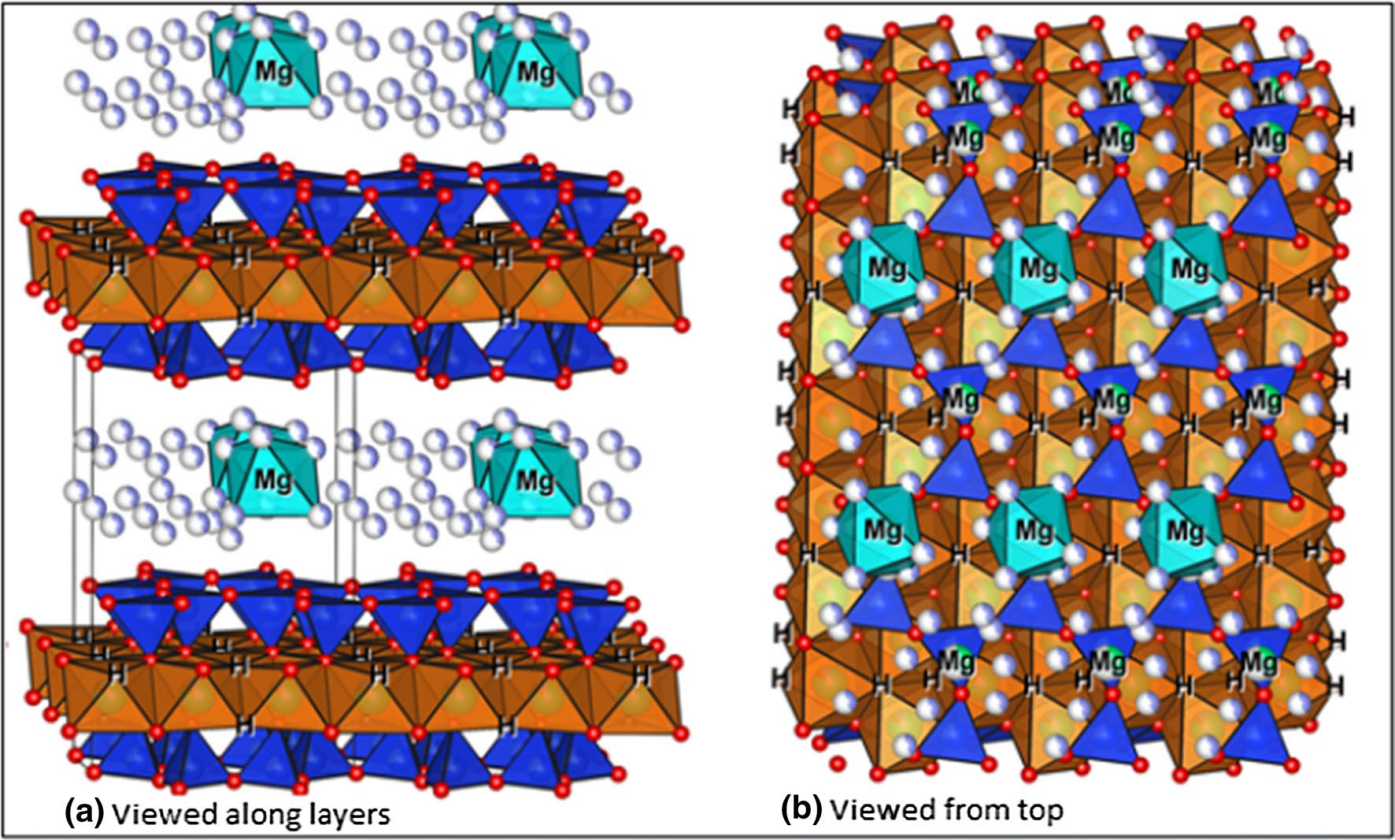
Simplified structure of layers of vermiculite generated using crystallographic data taken from Argüelles and co-workers [37]. Water molecules are shown as light purple spheres

As shown in Fig. 4, which was generated using crystallographic data from Argüelles and co-workers [37], vermiculite consists of talc-like silicate layers along the *a-b* plane, intercalated by hydrated magnesium ions. The solvated Mg^2+^ ions with an octahedral coordination of water molecules, are located between bases of [SiO_4_] tetrahedra from adjacent talc-type layers. Minerals with this kind of structure have weaker bonds between layers, which leads to the formation of different polytypes with the same motif, but slightly different relative positions of layers. The common feature of all these structures is the shortest distance between two identical layers, which is equal to ~ 14.3–15 Å. The layers shift relative to each other in a random way in directions (−*a/3,* + *b/3, 1*) or (−*a/3, *−*b/3, 1*). Furthermore, changes in chemical composition and humidity may lead to transformation of one structure into another [38]. The observations about the vermiculite structure suggest that this mineral, like talc, also has catalytic potential in the CO_2_ and epoxide cycloaddition reaction due to the presence of a number of active sites such as the -Mg-OH groups.

### CO_2_ and epoxide cycloaddition reaction

Initially, the activity of different phyllosilicates in the CO_2_ and epoxide cycloaddition reaction was tested in the absence of a co-catalyst, using propylene oxide (PO) as a model substrate. The results of the initial screening are presented in Table 2, and show no propylene carbonate (PC) formation for most of the catalysts, with the exception of talc and vermiculite, which produced PC in low yields. When the reaction was repeated under the same conditions but in the presence of a small amount of tetra-n-butylammonium bromide (TBAB) as a co-catalyst, an increase in activity for all catalysts in the coupling of CO_2_ with PO was observed. Under these experimental conditions, talc/ TBAB and vermiculite/ TBAB catalytic systems gave PO conversion of 68.7% and 60.3%, respectively. This suggested the necessity of a co-catalyst to facilitate the CO_2_ and epoxide cycloaddition process. Presence of an active solid support sometimes enhances the efficiency of a catalyst system through synergistic action, and absence of which may compromise catalytic performance [50]. The occurrence of synergistic effect in bifunctional catalyst systems consisting of inorganic metal oxides and Lewis bases has been previously observed for the cycloaddition process. Thus, for example, Whiteoak and co-workers [51], improved the cyclic carbonate yield from 0 to ≥ 75% when they used their catalyst in combination with quaternary ammonium halides. Also, Wang and co-workers [52], enhanced the performance of 3-hydroxypyridine catalyst from 6 to ≥ 82% in the presence of tetrabutylammonium iodide (TBAI) as co-catalyst.

**Table 2:** Conversion and selectivity in the coupling of propylene oxide (PO) and CO_2_ to produce propylene carbonate (PC) catalysed by various phyllosilicates at 120 °C

Entry	Catalyst	Co-catalyst	PO Conversion [%]	PC Selectivity [%]
1^a^	Biotite	–	Trace	–
2^a^	Chlorite	–	4.3	–
3^a^	Phlogopite	–	3.7	–
4^a^	Talc	–	10.2	27.5
5^a^	Vermiculite	–	8.6	30.1
6	Biotite	TBAB	44.5	68.3
7	Chlorite	TBAB	55.0	70.4
8	Phlogopite	TBAB	48.6	72.0
9	Talc	TBAB	68.7	94.2
10	Vermiculite	TBAB	60.3	96.1
11	Talc	TBAI	65.4	93.6
12	Talc	DMAP	36.9	84.7
13^b^	–	TBAB	42.0	96.8

Reaction conditions: propylene oxide (10 mmol), catalyst (100 mg), co-catalyst (2.5 mol % of epoxide), CO_2_ (20 bars), CH_3_CN (5 ml), Time (20 h)

^a^No co-catalyst

^b^No phyllosilicate

However, it should be noted that in this study, only small amounts of TBAB (2.5 mol % of the epoxide) was used, unlike in previous studies where a slightly higher catalyst loadings and/or higher reaction temperatures of 150 °C and pressures of 80 bars were utilized [24, 53, 54]. When TBAB was used as the only catalyst, the results were inferior (Table 2, entry 13) to those where both the phyllosilicate and co-catalyst were present, again indicating synergistic roles in the catalytic process. Nevertheless, it should be mentioned that quarternary ammonium salts (QAS) have been employed as sole catalysts in some homogeneous CO_2_ and epoxide coupling processes, although a high amount of the QAS was employed to achieve significant cyclic carbonate yields. For example, Caló and co-workers [55], used molten tetrabutylammonium halides as solvents and catalysts for the coupling of CO_2_ with various epoxides and reported yields in the range 10–90% under optimized reaction conditions.

Results of the initial screening also showed, that the performance of TBAB as a co-catalyst was slightly better than that of TBAI, an observation previously noted when utilizing similar catalytic systems [54]. On the other hand, when N, N-dimethylamino pyridine (DMAP) was used as co-catalyst, there was a reduction in both PO conversion and PC selectivity (Table 3, entry 12). This could be attributed to higher basicity of DMAP that might lead to formation of relatively stable intermediates hence affecting conversion and selectivity.

**Table 3:** Conversion and selectivity in the coupling of PO and CO_2_ to produce PC catalysed by talc and vermiculite under different conditions

Entry	Catalyst	Temperature	Pressure (Bars)	PO Conversion [%]	PC Selectivity [%]
1	Talc	100	20	59.3	94.4
2	Talc	100	30	60.5	94.6
3	Talc	120	30	68.7	94.2
4	Talc	130	30	87.6	94.3
5	Talc	140	10	97.5	93.6
6	Talc	140	20	98.0	93.9
7	Talc	140	30	98.9	93.7
8	Talc	140	35	99.0	93.4
9	Talc	150	30	99.3	92.2
10	Vermiculite	140	30	90.1	95.3
11	Vermiculite	150	30	92.6	93.7
12^a^	Talc	140	30	70.8	94.0
13^b^	Talc	140	30	99.2	92.8
14^c^	Talc	140	30	95.3	87.0
15^d^	Talc	140	30	96.8	92.4

Reaction conditions: propylene oxide (10 mmol), catalyst (100 mg), TBAB (2.5 mol % of epoxide), CO_2_ (10–35 bars), MeCN (5 ml), Temperature (100–150 °C), Time (20 h)

*PO* propylene oxide, *PC * propylene carbonate

^a^Time 12 h

^b^Time 24 h

^c^CH_2_Cl_2_ solvent

^d^DMF solvent

### Effect of reaction conditions

The effect of different reaction conditions such as temperature, pressure, reaction time and solvent were studied in order to improve conversion and selectivity of the CO_2_ and epoxide coupling catalyzed by phyllosilicates and TBAB. Talc and vermiculite that had exhibited superior performance in the cycloaddition reaction compared to other phyllosilicates were selected for further study and the results are presented in Table 3.

Temperature and CO_2_ pressure had a significant effect on the PO conversion whereas the PC selectivity was not particularly sensitive to these reaction conditions. The optimum temperature for the cycloaddition reaction was 140 °C with a pressure of 30 bars giving an overall PC yield of 93% (Table 2, entry 7). The effect of pressure on PO conversion was fairly small compared to the temperature effect and no improvement was seen above 30 bars. This observation is in agreement with earlier studies that reported no significant effect of pressure on the cycloaddition process in the range 30–100 bars [18, 54]. Furthermore, an increase in reaction time resulted into a higher conversion of PO, albeit with a slightly lower PC selectivity (Table 3, entry 7, 12 and 13). Results also showed that the effect of solvents was large with acetonitrile (MeCN) and dimethylformamide (DMF) giving a high PC selectivity, whereas dichloromethane (DCM) suppressed selectivity. Similar solvent effects have been previously reported [20, 56–58].

### Effect of heat pre-treatment of the catalysts

The effect of heat pre-treatment of the phyllosilicates on the catalytic activity was also studied and the results are presented in Table 4. Generally, the activity of the catalysts reduced slightly with an increase in calcination temperature (Table 4, entry 1 to 3), whereas the selectivity of the catalytic system slightly improved with heat pre-treatment of the phyllosilicates.

**Table 4:** Conversion and selectivity in the coupling of CO_2_ and PO to produce PC catalysed by phyllosilicates calcined at different temperatures

Entry	Catalyst	Calcination Temperature	PO Conversion [%]	PC Selectivity [%]
1	Talc	100	98.9	93.7
2	Talc	300	96.7	94.5
3	Talc	500	95.3	94.2
4	Vermiculite	100	90.1	95.3
5	Vermiculite	300	89.4	96.1
6	Vermiculite	500	87.6	96.4
7^[a]^	Talc	100	97.5	92.5
8^[a]^	Vermiculite	100	88.2	95.6
10^[b]^	Talc	100	96.5	92.8

Reaction conditions: propylene oxide (10 mmol), catalyst (100 mg), TBAB (2.5 mol % of epoxide), CO_2_ (30 bars), MeCN (5 ml), Temperature (140 °C), Time (20 h)

*PO* propylene oxide, *PC* propylene carbonate

^a^co-catalyst TBAI

^b^Talc catalyst dried at 100 °C and stored in a vial for 3 months

Prolonged storage of the ground phyllosilicates had a negative effect on their catalytic performance (Table 4, entry 10). The catalytic activity of the phyllosilicate minerals is derived from chemical behavior of the surface functional groups [59]. When the phyllosilicate layers are disturbed, during grinding, a diversity of functional groups is exposed. Since these groups were in the interior of the crystal, they become coordinatively unsaturated when exposed causing them to be very reactive [48]. However, when the active groups of the catalysts are exposed to the atmosphere for a long time, they adsorb moisture and other species which may affect their catalytic performance [60].

### Proposed mechanism for the cycloaddition of CO_2_ and PO using talc/ TBAB catalytic system

**Fig. 5:**
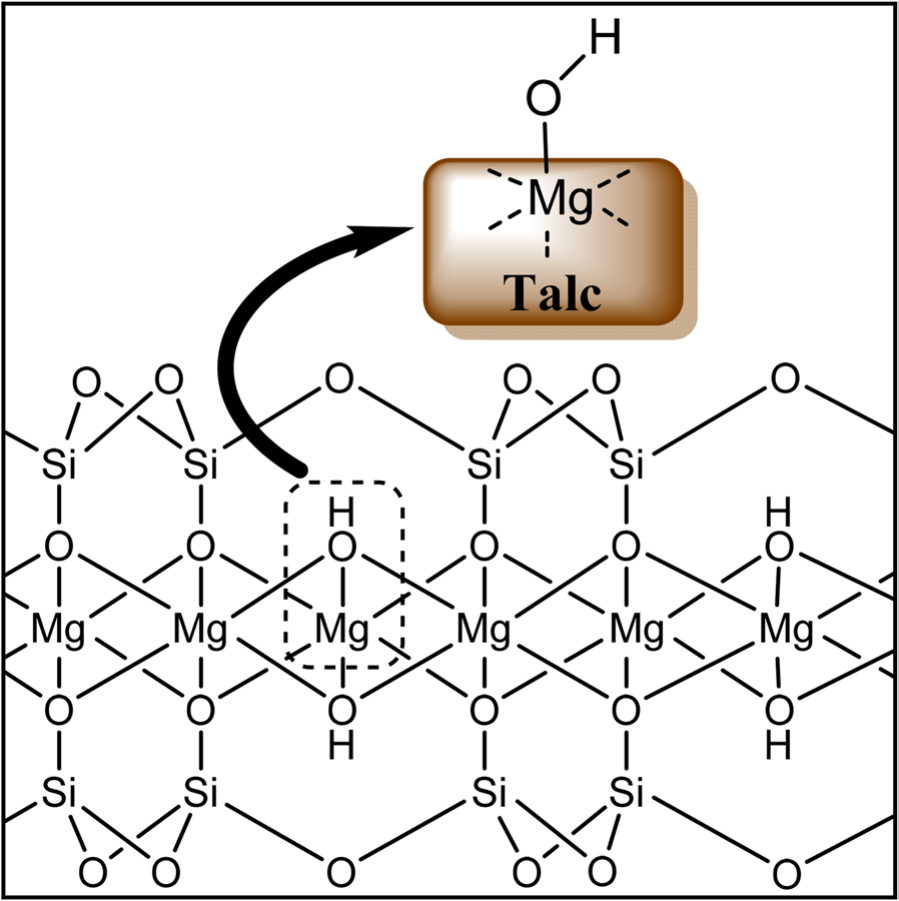
Simplified illustrated structure of talc showing the [MgO_6_] octahedral sheet sandwiched between two [SiO_4_] tetrahedral sheets, through shared oxygen atoms

Studies have shown that the most effective catalysts for the cycloaddition reaction contain Lewis-acid sites for epoxide activation and nucleophilic groups to promote ring-opening of the epoxide [61]. A simplified illustrated talc structure (Fig. 5), shows that each basal (001) layer is comprised of the [MgO_6_] octahedral sheet, sandwiched between two [SiO_4_] tetrahedral sheets through shared oxygen atoms [27, 62]. Various transition metal cations, including Fe^2+^, Fe^3+^, Ni^2+^, Ti^3+^, can be introduced into the octahedral sheet, whereas s-block metal cations, such as Na^+^, Ka^+^, Ca^2+^ and Mg^2+^, can also be introduced into the interlayers [27, 62]. Introduction of metals into the structure of talc implies that its catalytic properties are tunable. Based on the results obtained in the CO_2_ and epoxide cycloaddition reaction, and the structure of talc as illustrated in Fig. 5, we propose a hydrogen bond assisted mechanism for this process. Hydrogen bonding between the catalyst and epoxide is thought to be a key factor in promoting the cycloaddition reaction for this type of mechanism.

**Scheme 2:**
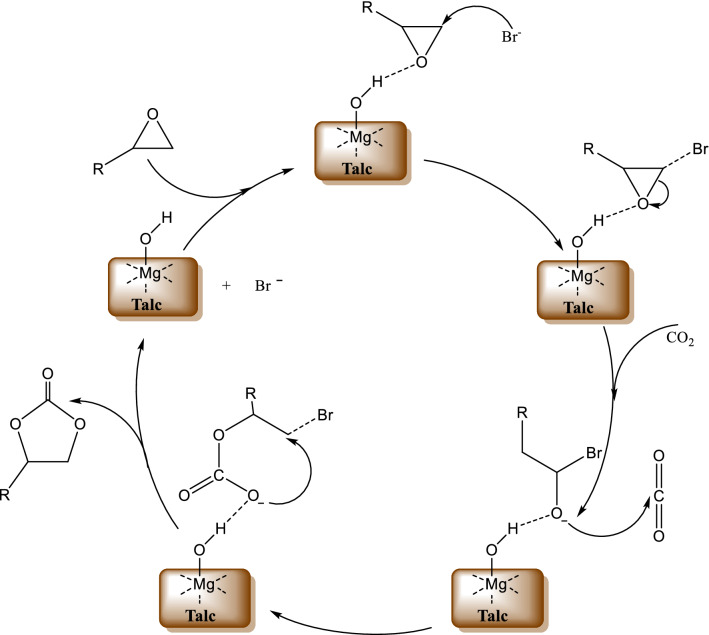
Proposed mechanism for the hydrogen bond assisted cycloaddition of CO_2_ and epoxides catalyzed by Talc/TBAB

In this work, talc or another phyllosilicate provides the Lewis-acid sites through the hydroxyl groups, while the co-catalyst (TBAB) provides the nucleophilic groups. As shown in Scheme 2, the hydroxyl group on the surface of talc acts as a weak acid, activating the epoxide by forming a hydrogen bond with it. The activated epoxide then undergoes a nucleophilic attack by Br- from the co-catalyst (TBAB), resulting in ring opening to form a halo-alkoxide. The oxygen anion of the ring-opened halo-alkoxide then interacts with CO_2_, which is activated by the Lewis acidic sites [63] to form an alkylcarbonate anion. The alkylcarbonate is transformed into a cyclic carbonate by intramolecular substitution of the Br- with the regeneration of TBAB. This mechanism is similar to that described for the cycloadditon of epoxides and CO_2_ using an iron-based composite catalyst rich in hydroxyl groups [22].

The results obtained using the heat pre-treated catalysts, further support the hydrogen bond assisted mechanism proposed for the CO_2_ and epoxide cycloaddition reaction. As shown in Table 4, an increase in calcination temperature in the range 100–500 °C resulted into a decrease in the activity of the catalysts (Table 4, entry 1–3). This might be considered contrary to what is expected since heating the phyllosilicates results in increased interlayer spacing which opens up the mineral structure. This would expose more potentially active sites resulting into enhanced catalytic activity, but in our study, a reduction in performance was observed. Thermal treatment of phyllosilicates in the temperature range 100–500 °C, does not cause significant structural or mineralogical decomposition of these minerals, but results in loss of water molecules [64, 65]. The structural decomposition of talc commences at ~ 800 °C, peaking at ~ 895 °C, with the formation of enstatite and amorphous silica [64]. Since the surface activity of the phyllosilicates is attributable to the presence of hydroxyl groups which coordinate and activate the epoxide through hydrogen bonding, loss of these groups as water molecules would result into a reduction in catalytic activity. These observations, provides more support for the proposed mechanism in Scheme 2, where the hydroxyl groups play a vital role in activating the epoxide.

Vermiculite also exhibited significant activity in the epoxide and carbon dioxide cycloaddition process, although its efficiency was slightly lower than that of talc. This is not surprising since vermiculite has some talc-like layers and contains Mg^2+^ and Al^3+^ ions, which are potentially active sites in the cycloaddition reaction. The reduction in catalytic activity of vermiculite compared to talc, could be because some of the active sites in the former are obscured by composition water. Indeed, water molecules, especially weakly bonded non-solvating ones, could compete with reagents for adsorption sites and hamper activity.

### CO_2_ coupling with various epoxides catalyzed by talc/TBAB

Basing on the interesting results obtained in the CO_2_ and PO coupling reaction using talc and TBAB under optimal conditions, different epoxides were also tested and the results are presented in Table 5. High conversion and selectivity to the corresponding cyclic carbonate was observed for epichlorohydrin, propylene oxide, butylene oxide and styrene oxide under the optimized reaction conditions (Table 5, entry 1–4). All cyclic carbonates were produced in ≥ 90% yields with the exception of butylene carbonate which was obtained in a slightly lower yield of 86.5%. The catalytic process could also be carried out with a reduced amount of the co-catalyst, although slightly lower cyclic carbonate yield was observed (Table 5, entry 7). After the catalytic runs, the phyllosilicate part of the catalyst system was recovered by filtration, washed, dried and re-used with a fresh amount of TBAB. The epoxide conversions and cyclic carbonate selectivities in the subsequent runs were similar to those obtained with fresh catalyst, suggesting that the catalyst could be used a number of times without significant loss in activity (Table 5, entry 5 and 6). The talc/ TBAB and vermiculite/TBAB catalyst systems in this study, exhibited good activity in the cycloaddition of CO_2_ with epoxides, producing the corresponding cyclic carbonates in high yields. Though some previous studies have reported medium to high yields of cyclic carbonates in the cycloaddition process utilizing comparable inorganic heterogeneous catalytic systems [22, 26, 66], the performance of these catalysts was challenged under solvent free conditions.

**Table 5:**
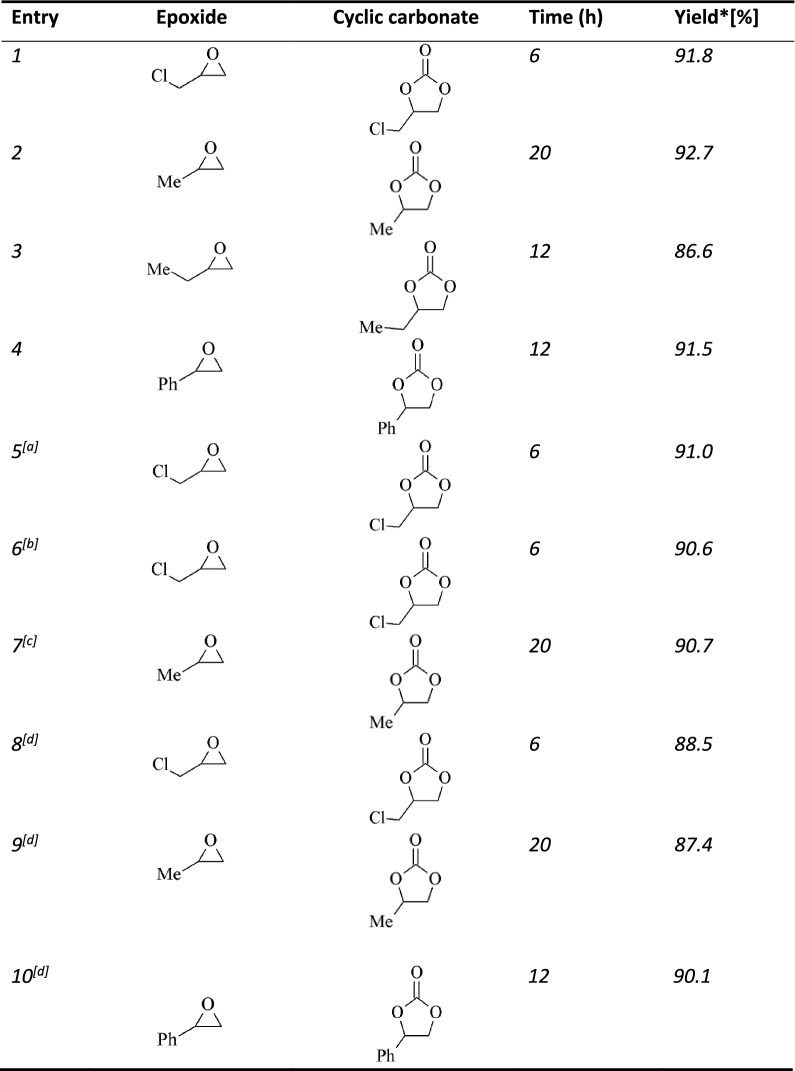
Conversion and selectivity in the coupling of various epoxides and CO_2_ catalysed by Talc dried at 100 °C

Reaction conditions:epoxide (10 mmol), Talc (100 mg), TBAB (2.5 mole% of epoxide), CO_2_ (30 bars), MeCN (5 ml), Temperature (140 °C)

Yield* = conversion x selectivity

^a^recycled catalyst

^b^twice recyled catalyst

^c^TBAB (2 mole% of epoxide). ^d^no solvent, epoxide (20 mmol), Talc (200 mg), TBAB (2.5 mole% of epoxide)

Avoiding use of solvents in chemical processing is a key goal in green chemistry. To this end, the CO_2_ and epoxide cycloaddition reaction was also carried out in the absence of a solvent, and the results are shown in Table 5. High conversions of ≥ 90% were obtained for a number of epoxides without loss of selectivity to the corresponding cyclic carbonate (Table 5; entry 8 to 10). A few studies have reported use of inorganic heterogenous catalysts for the cyclic carbonate synthesis in the absence of solvent, although most of the systems had conversion and selectivity challenges. Yamaguchi and co-workers [18], employed several metal oxides for the coupling of CO_2_ and PO to form PC in the absence of a solvent. The best oxide, gave a yield of 32% at 150 °C and 80 bars of CO_2_. Fujita and co-workers [24], used smectite based catalysts containing various amounts of alkali metals in the cycloaddition of CO_2_ and PO without a solvent. The most active smectite catalyst gave a PC yield of 80% at 150 °C and 80 bars of CO_2_. Srivastava and co-workers [26], reported a PC yield of 86.3% when utilizing zeolite based organic–inorganic hybrid catalysts for the CO_2_ and epoxide coupling under solvent free conditions. The present catalyst system offers a PC yield of 87.4% in the absence of a solvent, which is slightly higher than the systems previously reported and mentioned above. Furthermore, the corresponding cyclic carbonates for other epoxides such as epichlorohydrin and styrene oxide were also produced in high yields under solvent-free conditions. Therefore, the results of this study, ably demonstrate the catalytic potential of talc and vermiculite in the cycloaddition of CO_2_ and epoxide to form cyclic carbonates in excellent yields, despite need for a co-catalyst. The low cost and availability of naturally occurring talc and other phyllosilicate deposits, makes this strategy attractive for the cycloaddition process.

## Conclusion

We have successfully demonstrated the use of talc and other physllosilicates as tunable catalysts for the direct synthesis of cyclic carbonates from CO_2_ and epoxide. These naturally occurring materials show high activity in the CO_2_ and epoxide coupling reaction in the presence of TBAB as a co-catalyst producing the corresponding cyclic carbonates in excellent yields and selectivity. The phyllosilicate part of the catalysts is easily recovered by filtration and can be reused several times. The catalyst system can also work efficiently under solvent-free conditions. These naturally occurring minerals are low cost, stable and efficient catalysts for the cycloaddition process with potential for further exploration.

## Experimental section

### General considerations

Commercially available solvents and reagents were used as received. The natural vermiculite samples were obtained from Namekhara mines, Manafwa District, Eastern Uganda. The natural talc samples were obtained from Lolung-Moruamakale deposits in Moroto District, Northern Uganda. Other phyllosilicates, were selected from voucher mineral samples at the Department of Geology and Petroleum studies, College of Natural Sciences, Makerere University. The elemental composition of the samples was determined using ICP on a PerkinElmer OPTIMA 3000 DV Instrument. The IR spectra of the catalysts were recorded on a Shimadzu FTIR 8201 PC instrument using KBr discs. Gas chromatography analyses were done with a Hewlett Packard 5890 Series II gas chromatograph equipped with an FID detector. GC–MS studies were performed on an Agilent 6890 N instrument. The surface area, average adsorption pore size and volume for the talc catalysts were measured by Nitrogen gas analysis using a Micromeritics ASAP 2420 instrument.

### Catalyst preparation and pretreatment

Naturally occurring phyllosilicates including talc, vermiculite, chlorite, phlogopite and biotite were selected for the study. The samples were dried at 100 °C, crushed in a mortar, and the resultant catalyst powder stored in tightly closed sample bottles. Some of the samples were calcined at different temperature in the range 300–500 °C before being tested in the cycloaddition process. Talc and vermiculite showed the most promising results in catalysis and were fully characterized using different techniques whereas the other phyllosilicates were used without any further characterization. Muscovite was not included in the study because of its sheet like nature.

### Powder X-ray diffraction

X-ray powder diffraction data for the mineral samples was collected in transmission mode using a Stoe Stadi MP diffractometer (Cu Kα1, λ = 1.54051 Å, detector Mythen 1 K, step size 0.015°). Samples of pristine minerals were gently crushed in mortar to small particles of ~ 0.1–0.2 mm size but without excessive comminution, in order to avoid extensive structure distortion. The talc powder was put between two sticky layers of scotch in a flat sample holder for the measurement in θ–2θ mode (4 runs were merged, 2θ = 2–116°, 5° PSD step, 40 s/PSD step). The powder of vermiculite, consisting of thin platelets, was measured in a rotating glass capillary with diameter 0.3 mm (5 runs were merged, 2θ = 2–121°, 5° PSD step, 420 s/PSD step) in Debye–Scherrer mode. The phase composition of samples was determined by comparison of the powder diffraction data with powder patterns of known phases using the program package Stoe WinXPOW [37, 45, 49].

### Catalytic reactions

The CO_2_ and epoxide cycloaddition reactions were performed in a 100 mL stainless steel autoclave equipped with a magnetic stirrer. The epoxide (10–20 mmol), catalyst (0–200 mg), co-catalyst (0–2.5 mol % of epoxide) and solvent (0–5 ml) were loaded into the autoclave. The autoclave was purged with CO_2_ and then pressurized with an appropriate amount of CO_2_ (10–35 bar). The reaction was carried out at the desired temperature (100–150 °C) for a duration of 4–24 h with constant stirring. At the end of the reaction, the reactor was cooled in an ice-water bath, and the unreacted CO_2_ released slowly. All catalytic runs were performed in duplicates and the average result evaluated. The products were analysed using a combination of GC-FID and GC–MS with reference to an internal standard (biphenyl) which was added to the mixture before the reaction. Where necessary, the cyclic carbonates were obtained from the reaction mixture using distillation or recrystallization from ethanol [67].

## Supplementary information

**Additional file 1: Figure S1.** FTIR Spectrum of talc. **Figure S2.** N_2_ Adsorption–desorption isotherm.

## Data Availability

All supporting information including figures and detailed methods is available upon request.

## Abbreviations

CAS: Centre for Analysis and Synthesis
CO_2_: Carbon dioxide
DCM: Dichloromethane
DMAP: N, N-dimethylamino pyridine
DMF: Dimethylformamide
FID: Flame ionization detector
GC: Gas chromatography
IPICS: International Programme in Chemical Sciences
ISP: International Science Programme
MS: Mass spectrometry
OPCW: Organization for Prohibition of Chemical Weapons
PC: Propylene carbonate
PO: Propylene oxide
PXRD: Powder X-ray diffraction
QAS: Quarternary ammonium salts
TBAB: N-tetra butyl ammonium bromide
TBAI: N-tetra butyl ammonium iodide
