# Supplementary material for: Synthesis of cyclic carbonates from epoxides and carbon dioxide catalyzed by talc and other phyllosilicates
Source: BMC Chem. 2020 Oct 20;14(1):61. doi: 10.1186/s13065-020-00713-2 (PMC7576757; doi:10.1186/s13065-020-00713-2)
Supplement: Supplementary file 1 — Additional file 1: Figure S1. FTIR Spectrum of talc. Figure S2. N2 Adsorption–desorption isotherm. [file 13065_2020_713_MOESM1_ESM.docx]

**Synthesis of cyclic carbonates from epoxides and carbon dioxide catalyzed by**

**talc and other phyllosilicates**

Fiona Nakibuule^1,2^, Steven Allan Nyanzi^1^, Igor Oshchapovsky^2,3^, Ola F. Wendt^2^ and Emmanuel Tebandeke^1^*

**Additional file 1**


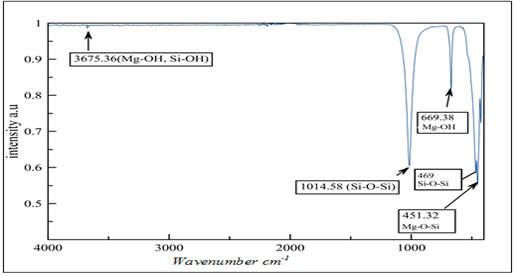


Figure S 1. FTIR spectrum of talc catalyst.


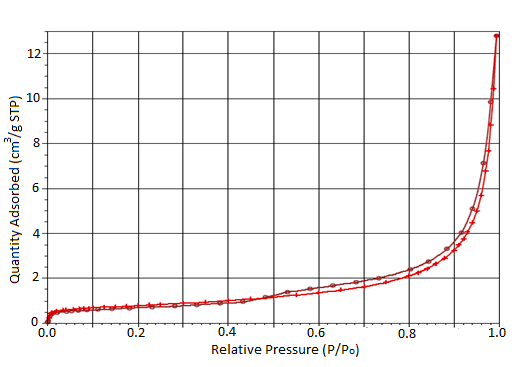


Figure S 2. N_2_ Adsorption-desorption Isotherm for talc.
